# Temporally and Spatially Localized PKA Activity within Learning and Memory Circuitry Regulated by Network Feedback

**DOI:** 10.1523/ENEURO.0450-21.2022

**Published:** 2022-04-01

**Authors:** James C. Sears, Kendal Broadie

**Affiliations:** 1 Department of Biological Sciences, Vanderbilt University and Medical Center, Nashville, TN 37235; 2 Vanderbilt Brain Institute, Vanderbilt University and Medical Center, Nashville, TN 37235; 3 Department of Cell and Developmental Biology, Vanderbilt University and Medical Center, Nashville, TN 37235; 4 Department of Pharmacology, Vanderbilt University and Medical Center, Nashville, TN 37235

**Keywords:** *Drosophila*, FMRP, Kenyon cell, Meng-Po, mushroom body, neurobeachin

## Abstract

Dynamic functional connectivity within brain circuits requires coordination of intercellular signaling and intracellular signal transduction. Critical roles for cAMP-dependent protein kinase A (PKA) signaling are well established in the *Drosophila* mushroom body (MB) learning and memory circuitry, but local PKA activity within this well-mapped neuronal network is uncharacterized. Here, we use an *in vivo* PKA activity sensor (PKA-SPARK) to test spatiotemporal regulatory requirements in the MB axon lobes. We find immature animals have little detectable PKA activity, whereas postcritical period adults show high field-selective activation primarily in just 3/16 defined output regions. In addition to the age-dependent PKA activity in distinct α’/β’ lobe nodes, females show sex-dependent elevation compared with males in these same restricted regions. Loss of neural cell body Fragile X mental retardation protein (FMRP) and Rugose [human Neurobeachin (NBEA)] suppresses localized PKA activity, whereas overexpression (OE) of MB lobe PKA-synergist Meng-Po (human SBK1) promotes PKA activity. Elevated Meng-Po subverts the PKA age-dependence, with elevated activity in immature animals, and spatial-restriction, with striking γ lobe activity. Testing circuit signaling requirements with temperature-sensitive *shibire* (human Dynamin) blockade, we find broadly expanded PKA activity within the MB lobes. Using transgenic tetanus toxin to block MB synaptic output, we find greatly heightened PKA activity in virtually all MB lobe fields, although the age-dependence is maintained. We conclude spatiotemporally restricted PKA activity signaling within this well-mapped learning/memory circuit is age-dependent and sex-dependent, driven by FMRP-Rugose pathway activation, temporally promoted by Meng-Po kinase function, and restricted by output neurotransmission providing network feedback.

## Significance Statement

Learning and memory requires the coordination of cell-cell circuit interactions with the appropriate downstream signal transduction. Plasticity regulation via cAMP-dependent protein kinase A (PKA) activation is among the best-characterized signaling pathways, but until recently, it was very challenging to localize PKA signaling within brain circuits. We employ a new PKA biosensor to visualize local signaling in the *Drosophila* mushroom body (MB); a network model consolidating ∼2000 Kenyon cells (KCs) into ∼16 output fields per brain hemisphere. We discover heightened PKA activity in just a few circuit regions, with age-dependent and sex-dependent requirements. Learning/memory mutants linked to intellectual and autism spectrum disorders (ASDs) reduce PKA activity. In contrast, PKA-synergist Meng-Po increases PKA activity. Neurotransmission blockade elevates and expands PKA activity, showing network feedback restricts localized signaling.

## Introduction

Functional connectivity and plasticity within brain circuits requires orchestration of intercellular network interactions and their downstream intracellular signaling cascades. For learning acquisition and memory consolidation, cAMP activation of protein kinase A (PKA) is widely recognized to be required for this synaptic regulation (Nguyen and Woo, 2003; Khan et al., 2021). In the *Drosophila* mushroom body (MB) learning/memory circuit model, both PKA and PKA regulators (e.g., Rutabaga adenylyl cyclase, Dunce phosphodiesterase) are highly expressed, and play well-characterized, pivotal roles (Nighorn et al., 1991; Crittenden et al., 1998; Lee, 2015). Within each brain hemisphere, ∼2000 MB Kenyon cells (KCs) project into five distinct neuropil lobes (α/β, α’/β’, and γ) to innervate 16 defined MB output neuron (MBON) regions with en passant connectivity (Aso et al., 2014b). MB lobes receive layers of dopaminergic, GABAergic and serotonergic synaptic feedback, which provide context and regulate the KC output (Scheunemann et al., 2018; Modi et al., 2020; Yamagata et al., 2021). Investigations of α/β and γ MB lobes in immobilized animals reveal low basal PKA activity, with pharmacological treatment differentially activating PKA function dependent on circuit location (Gervasi et al., 2010). However, a lack of visualization tools has limited studies of local PKA activity within the MB lobes, until the recent development of a PKA activity biosensor (PKA-SPARK), which generates bright GFP puncta on PKA phosphorylation (Zhang et al., 2018; Sears et al., 2019; Sears and Broadie, 2020). New studies of PKA-SPARK signaling within the increasingly well-defined MB circuit promise to provide critical insights into learning/memory impairments.

Fragile X mental retardation protein (FMRP) and Rugose/Neurobeachin (NBEA) are causally linked to intellectual disability and autism spectrum disorder (ASD; Wang et al., 2000; Castermans et al., 2003, 2010; Harris et al., 2008). FMRP drives cAMP induction and PKA activation in Fragile X syndrome (FXS) mouse/*Drosophila* disease models and human FXS patient cell cultures lacking FMRP (Berry-Kravis et al., 1995; Kelley et al., 2007; Sears and Broadie, 2020), and FMRP loss-of-function (LOF) impairs both learning and memory (Bolduc et al., 2008). FMRP binds *rugose* mRNA to positively regulate translation of this A-kinase anchor protein (AKAP) controlling MB PKA activity (Sears et al., 2019), and Rugose LOF also causes defective MB-dependent learning and memory (Volders et al., 2012; Buddika et al., 2021). Downstream of PKA, Meng-Po kinase [human SH3-binding kinase 1 (SBK1); Nara et al., 2001] is expressed within KC axons in the *Drosophila* MB lobes to facilitate memory formation (Lee et al., 2018). Meng-Po/SBK1 overexpression (OE) in the MB strengthens long-term memory formation. To test MB intercellular circuit signaling requirements, the temperature-sensitive *shibire^ts^* mutant blocking Dynamin-dependent synaptic vesicle (SV) endocytosis can be used to disrupt KC neurotransmission (Koenig et al., 1983; Kitamoto, 2001). The transgenic tetanus toxin light chain (TNT) protease blocks SV fusion to more comprehensively eliminate KC neurotransmission (Sweeney et al., 1995; Haag et al., 2016). Our aim in this study was to test MB lobe PKA activity with all of the above intercellular and intracellular signaling perturbations, employing the transgenic PKA-SPARK reporter (Zhang et al., 2018) to visualize spatiotemporal signaling regulation.

In this study, we compare immature *Drosophila* at 0 d posteclosion (0 dpe) with postcritical period *Drosophila* adults (7 dpe). Immature animals display little or no detectable PKA-SPARK activity in the MB lobes, whereas mature animals exhibit high levels of spatially-restricted signaling in α’1 and β’1 regions. In both locations, we find females have higher PKA activity. FMRP, Rugose and Meng-Po loss results in strongly reduced PKA activity in both MB lobe regions. In contrast, Meng-Po OE drives PKA activity in immature animals (0 dpe), as strongly as normal mature signaling, and also dramatically increases PKA activity in α’1, β’1, and β’1-adjacent γ3 at maturity (7 dpe). Testing circuit signaling requirements, we targeted KC synaptic output with two tools; temperature-sensitive *shibire^ts^* and TNT blockade. The *shibire^ts^* animals display both age-dependent and temperature-dependent defects in PKA-SPARK activity, with expanded PKA signaling within the MB lobes at restrictive temperature. Consistently, MB-targeted TNT neurotransmission blockade dramatically expands PKA activity throughout the lobes, although the age-dependence is maintained. These findings indicate that PKA activity in the MB lobes is both age-dependent and sex-dependent in tightly restricted connectivity regions. Moreover, PKA activity is selectively induced during early adulthood in a mechanism requiring FMRP, Rugose, and Meng-Po and can be strongly potentiated by elevated Meng-Po to overcome some spatiotemporal restrictions. Finally, synaptic output suppresses PKA activity to spatially defined MB lobe regions of heightened signaling, suggesting a network feedback mechanism regulates PKA activity.

## Materials and Methods

### *Drosophila* genetics

All animals were reared and maintained on standard food in 12/12 h light/dark cycle cycling incubators at 25°C, unless otherwise noted. A combination of females and males were used for all experiments, except when females and males were compared or mutations were on the X chromosome. For mutant studies, the genetic background *w^1118^* (BDSC 3605) and RNAi background (P{y[+t7.7]=CaryP}attP2; BDSC 36303) were used as controls. Null *dfmr1^50M^* (Zhang et al., 2001) and *rg^FDD^* (Volders et al., 2012) mutants were assayed in the *w^1118^* background. For transgenic studies, *w^1118^* outcrossed to the OK107-Gal4 driver line (BDSC 854) was used as the control. The OK107-Gal4 driver line at 2 dpe was used as a negative control for Western blot analyses. UAS responder lines used included: UAS-PKA-SPARK (Zhang et al., 2018), UAS-*dfmr1* (Zhang et al., 2001), UAS-*hFMRP^ISO7^* (Sears and Broadie, 2020), UAS*-ΔRGG-hFMRP* (Coffee, 2011), UAS-*rg* (Volders et al., 2012), UAS-*meng-po* (Lee et al., 2018), UAS-*meng-po* RNAi (BDSC 29603; Lee et al., 2018), UAS-*shibire^ts^* (BDSC 44222), and insulated UAS-TNT (a generous gift from Brian McCabe; Brain Mind Institute, EPFL, Switzerland). For acute *shibire^ts^* experiments, animals were raised at 20°C until 7 dpe, and then either maintained at 20°C or moved to 33°C for 3 h. For longer *shibire^ts^* experiments, animals were raised at 20°C, and then either maintained at this temperature or moved to 25°C or 33°C for 7 d. Recombination and multiallele crossing schemes were performed using standard *Drosophila* genetic techniques.

**Figure 1.**
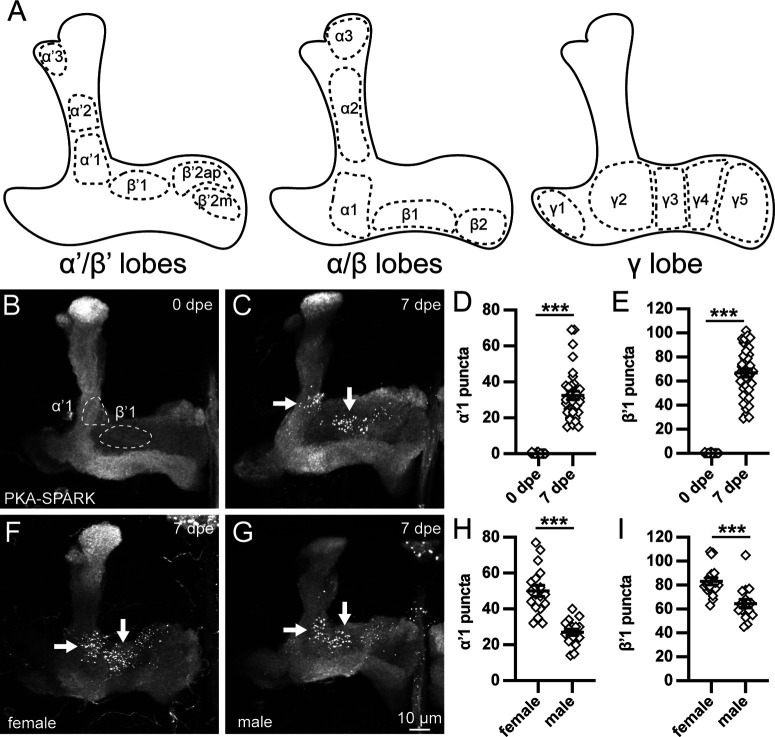
Early life, sex-dependent PKA activity in Mushroom Body circuit regions. ***A***, Schematic of MB lobes and defined MBON fields (dashed outlines) shown in three layers: α’/β’ (left), α/β (middle), and γ lobes (right). ***B***, ***C***, Representative images of MB lobes with OK107-Gal4 driving UAS-PKA-SPARK at 0 dpe (***B***) and 7 dpe (***C***). The MBON fields (dashed circles) and arrows delineate the α’1 and β’1 quantified regions. ***D***, ***E***, PKA-SPARK::GFP puncta number in both regions, including α’1 (***D***) and β’1 (***E***). Scatter plots show all data points and mean ± SEM. ***F***, ***G***, MB lobes with OK107-Gal4 driving UAS-PKA-SPARK in female (***F***) and male (***G***) at 7 dpe. ***H***, ***I***, Quantification of PKA-SPARK puncta in both α’1 (***H***) and β’1 (***I***). Sample size >15 fields in all conditions. Statistics show two-tailed *t* tests with Welch’s correction (***H***) or Mann–Whitney tests (***D***, ***E***, ***I***). Significance: ***p <* *0.001.

### Brain imaging

Staged brains were dissected free in PBS and fixed in 4% paraformaldehyde in PBS + 4% sucrose for 30 min with rotation. Fixed brains were then washed 3× in PBS, and incubated for 1.5 h in blocking buffer (PBS, 1% BSA, 0.5% goat serum, 0.2% Triton X-100). Samples were then incubated 2 h at room temperature (RT) with FITC-conjugated goat anti-GFP (1:500; Abcam, ab6662), and washed 3× in blocking buffer. Brain preparations were mounted in Fluoromount-G between two #1 coverslips separated by die-cut Oracal 651 spacers. All imaging was done using a Zeiss LSM510 META with a Plan Neofluar 40× oil-immersion (1.3 NA) objective. MB images were collected at 1-μm optical slice thickness for data analyses.

### Western blottings

Heads from staged animals (0 or 7dpe, female or male) were snap frozen on dry ice and then homogenized in 12-μl 1 × PBS with EDTA-free protease inhibitor (Roche; 04693132001). For each lysate, two pooled heads in 24-μl RIPA buffer (Sigma; R0278-50ML) were spun (16,000 × *g*) for 10 min at 4°C. Supernatant was removed and treated with λ Protein Phosphatase following the manufacturer protocol (New England Biolabs; P0753S). To equal lysate volumes, 2 × Laemmli (Bio-Rad; 161-0737) + 2-mercaptoethanol (Sigma-Aldrich; M6250-100ML) was added and samples heated to 100°C for 10 min. Precision Plus Protein All Blue Standards (Bio-Rad; 1610373) and samples were loaded into 4–15% Mini-PROTEAN TGX Stain-Free Gels (Bio-Rad; 4568086), and run at 100V for 10 min, then 180–200 V until completion. Protein was transferred onto Bio-Rad LF PVDF membranes using the Bio-Rad Trans-Blot Turbo system. Membranes were blocked in LI-COR Intercept Blocking Buffer (927-60001) at RT for 1 h, incubated in primary antibodies at RT for 1 h and 10 min, and fluorescent secondary antibodies at RT for 40 min. Labeled membranes were imaged on a LI-COR Odyssey CLx system. Protein bands were standardized to loading control (α-tubulin) and analyzed using ImageStudioLite (LI-COR). Primary antibodies: mouse anti-α-tubulin (DSHB; 12G10; 1:10,000 or 1:20,000) and rabbit anti-GFP (Abcam; ab290; 1:5000). Fluorescent secondary antibodies: Alexa Fluor 700 goat anti-rabbit (Invitrogen; A21038) and DyLight 800 goat anti-mouse (Invitrogen; SA535521), both at 1:10,000.

### Data analyses

All image analyses were conducted blind using ImageJ. PKA-SPARK puncta were counted using the ImageJ Point Tool. All statistical analyses were conducted using GraphPad Prism (v9). Datasets passing the D’Agostino and Pearson normality test were compared with two-tailed Welch’s *t* tests (*n* = 2 datasets) or Brown–Forsythe and Welch ANOVA tests (*n* > 2 datasets). Datasets failing normality tests were compared with Mann–Whitney tests (*n* = 2 datasets) or with Kruskal–Wallis tests with Dunn’s correction (*n* > 2 datasets). Graphs display all individual data points and mean ± SEM. Significance is displayed in the figures as *p <* *0.05, **p <* *0.005, ***p <* *0.001, and not statistically significant (*p *>* *0.05; n.s.). Additional information is presented in the statistical table (Table 1).

**Table 1:** Statistical tests used to analyze data

Circuit location	Comparison	Data structure	Test	Sample size (# KC fields or Western lysates)	Mean	Statistics	*p* value
α’1	0 dpe; 7 dpe	Not normal	Mann–Whitney test	38; 34	0.1; 32.5	*U = 0*	*p *<* *0.0001
β’1	0 dpe; 7 dpe	Not normal	Mann–Whitney test	37; 38	0.1; 66.8	*U = 0*	*p *<* *0.0001
α’1	Female 7 dpe; male 7 dpe	normal	Unpaired two-tailed *t* test	18; 16	50.1; 26.9	*t *=* *6.51 df = 26.96	*p *<* *0.0001
β’1	Female 7 dpe; male 7 dpe	Not normal	Mann–Whitney test	18; 16	83.2; 64.5	*U = 31*	*p *<* *0.0001
N/A	Female 0 dpe; male 0 dpe	Normal	Brown–Forsythe, Welch ANOVA test	9; 9	1.0; 1.104	*t *=* *2.028 DF = 15.75	*p *=* *0.2846
N/A	Female 0 dpe; female 7 dpe	Normal	Brown–Forsythe, Welch ANOVA test	9; 9	1.0; 1.088	*t *=* *0.8219 DF = 10.31	*p *=* *0.9482
N/A	Female 0 dpe; male 7 dpe	Normal	Brown–Forsythe, Welch ANOVA test	9; 9	1.0; 0.9298	*t *=* *0.7746 DF = 11.32	*p *=* *0.9608
N/A	Male 0 dpe; female 7 dpe	Normal	Brown–Forsythe, Welch ANOVA test	9; 9	1.104; 1.088	*t *=* *0.1493 DF = 9.809	*p *>* *0.9999
N/A	Male 0 dpe; male 7 dpe	Normal	Brown–Forsythe, Welch ANOVA test	9; 9	1.104; 0.9298	*t *=* *1.956 DF = 10.63	*p *=* *0.3383
N/A	Female 7 dpe; male 7 dpe	Normal	Brown–Forsythe, Welch ANOVA test	9; 9	1.088; 0.9298	*t *=* *1.223 DF = 15.43	*p *=* *0.7724
α’1	Control 7dpe; *dfmr1* 7dpe	Normal	Unpaired two-tailed *t* test	34; 21	43.5; 26.1	*t *=* *3.77 df = 41.34	*p *=* *0.0005
β’1	Control 7dpe; *dfmr1* 7dpe	Normal	Unpaired two-tailed *t* test	34; 21	75.5; 43.9	*t *=* *3.88 df = 34.73	*p *=* *0.0004
α’1	Control 7dpe; *rg* 7 dpe	Not normal	Mann–Whitney test	24; 18	27.4; 15.2	*U *=* *70.50	*p *=* *0.0001
β’1	Control 7dpe; *rg* 7 dpe	Normal	Unpaired two-tailed *t* test	24; 18	58.9; 20.8	*t *=* *8.40 df = 31.59	*p *<* *0.0001
α’1	Control 0 dpe; control 7 dpe	Not normal	Mann–Whitney test	14; 12	0.1; 35.7	*U = 0*	*p *<* *0.0001
α’1	dFMRP OE 0 dpe; dFMRP OE 7 dpe	Not normal	Mann–Whitney test	16; 18	5.2; 31.6	*U *=* *11.50	*p *<* *0.0001
α’1	hFMRP OE 0 dpe; hFMRP OE 7 dpe	Not normal	Mann–Whitney test	18; 11	0.2; 39.8	*U = 0*	*p *<* *0.0001
α’1	ΔRGG-hFMRP OE 0 dpe; ΔRGG-hFMRP OE 7 dpe	Not normal	Mann–Whitney test	8; 18	0.1; 5.3	*U *=* *9.50	*p *=* *0.0001
α’1	Rg OE 0 dpe; Rg OE 7 dpe	Normal	Unpaired two-tailed *t* test	14; 16	0.3; 31.6	*t *=* *8.94 df = 15.04	*p *<* *0.0001
α’1	Meng-Po OE 0 dpe; Meng-Po OE 7dpe	Normal	Unpaired two-tailed *t* test	16; 16	67.1; 196.4	*t* = 7.88 df = 16.78	*p *<* *0.0001
α’1	Control 0 dpe; dFMRP OE 0 dpe	Not normal	Kruskal–Wallis test	14; 16	0.1; 5.2	Mean rank diff = −22.9	*p *=* *0.0272
α’1	Control 0 dpe; hFMRP OE 0 dpe	Not normal	Kruskal–Wallis test	14; 18	0.1; 0.2	Mean rank diff = −0.7143	*p *>* *0.9999
α’1	Control 0 dpe; ΔRGG-hFMRP OE 0 dpe	Not normal	Kruskal–Wallis test	14; 8	0.1; 0.1	Mean rank diff = 0.5357	*p *>* *0.9999
α’1	Control 0 dpe; Rg OE 0 dpe	Not normal	Kruskal–Wallis test	14; 14	0.1; 0.3	Mean rank diff = −4.286	*p *>* *0.9999
α’1	Control 0 dpe; Meng-Po OE 0 dpe	Not normal	Kruskal–Wallis test	14; 16	0.1; 67.1	Mean rank diff = −49.21	*p *<* *0.0001
α’1	Control 7 dpe; dFMRP OE 7 dpe	Normal	Brown–Forsythe, Welch ANOVA test	12; 18	35.7; 31.6	*t *=* *0.74 DF = 23.56	*p *=* *0.9505
α’1	Control 7 dpe; hFMRP OE 7 dpe	Normal	Brown–Forsythe, Welch ANOVA test	12; 11	35.7; 39.8	*t *=* *0.62 DF = 20.17	*p *=* *0.9751
α’1	Control 7 dpe; ΔRGG-hFMRP OE 7 dpe	Normal	Brown–Forsythe, Welch ANOVA test	12; 18	35.7; 5.3	*t *=* *6.81 DF = 12.48	*p *<* *0.0001
α’1	Control 7 dpe; Rg OE 7 dpe	Normal	Brown–Forsythe, Welch ANOVA test	12; 16	35.7; 31.6	*t *=* *0.74 DF = 22.91	*p *=* *0.9508
α’1	Control 7 dpe; Meng-Po OE 7 dpe	Normal	Brown–Forsythe, Welch ANOVA test	12; 16	35.7;196.4	*t *=* *9.74 DF = 17.16	*p* < 0.0001
β’1	Control 0 dpe; control 7 dpe	Not normal	Mann–Whitney test	14; 12	0.1; 53.6	*U *=* *0	*p *<* *0.0001
β’1	dFMRP OE 0 dpe; dFMRP OE 7 dpe	Not normal	Mann–Whitney test	16; 18	3.4; 19.6	*U *=* *48.50	*p *=* *0.0005
β’1	hFMRP OE 0 dpe; hFMRP OE 7 dpe	Not normal	Mann–Whitney test	18; 11	1.0; 60.5	*U *=* *1	*p *<* *0.0001
β’1	ΔRGG-hFMRP OE 0 dpe; ΔRGG-hFMRP OE 7 dpe	Not normal	Mann–Whitney test	8; 18	0.1; 7.1	*U *=* *0	*p *<* *0.0001
β’1	Rg OE 0 dpe; Rg OE 7 dpe	Normal	Unpaired two-tailed *t* test	14; 16	0.4; 25.7	*t *=* *9.12 df = 15.12	*p *<* *0.0001
β’1	Meng-Po OE 0 dpe; Meng-Po OE 7dpe	Normal	Unpaired two-tailed *t* test	16; 22	138.9; 251.9	*t *=* *7.49 df = 26.44	*p *<* *0.0001
β’1	Control 0 dpe; dFMRP OE 0 dpe	Not normal	Kruskal–Wallis test	14; 16	0.1; 3.4	Mean rank diff = −18.48	*p *=* *0.1195
β’1	Control 0 dpe; hFMRP OE 0 dpe	Not normal	Kruskal–Wallis test	14; 18	0.1; 1.0	Mean rank diff = −6.127	*p *>* *0.9999
β’1	Control 0 dpe; ΔRGG-hFMRP OE 0 dpe	Not normal	Kruskal–Wallis test	14; 8	0.1; 0.1	Mean rank diff = −1.634	*p *>* *0.9999
β’1	Control 0 dpe; Rg OE 0 dpe	Not normal	Kruskal–Wallis test	14; 14	0.1; 0.4	Mean rank diff = −9.179	*p *>* *0.9999
β’1	Control 0 dpe; Meng-Po OE 0 dpe	Not normal	Kruskal–Wallis test	14; 16	0.1; 138.9	Mean rank diff = −50.82	*p *<* *0.0001
β’1	Control 7 dpe; dFMRP OE 7 dpe	Normal	Brown–Forsythe, Welch ANOVA test	12; 18	53.6; 19.6	*t *=* *5.13 DF = 20.78	*p* = 0.0002
β’1	Control 7 dpe; hFMRP OE 7 dpe	Normal	Brown–Forsythe, Welch ANOVA test	12; 11	53.6; 60.5	*t *=* *0.76 DF = 19.17	*p *=* *0.9426
β’1	Control 7 dpe; ΔRGG-hFMRP OE 7 dpe	Normal	Brown–Forsythe, Welch ANOVA test	12; 18	53.6; 7.1	*t *=* *8.31 DF = 12.07	*p *<* *0.0001
β’1	Control 7 dpe; Rg OE 7 dpe	Normal	Brown–Forsythe, Welch ANOVA test	12; 16	53.6; 25.7	*t *=* *4.55 DF = 16.56	*p* = 0.0014
β’1	Control 7 dpe; Meng-Po OE 7 dpe	Normal	Brown–Forsythe, Welch ANOVA test	12; 22	53.6; 251.9	*t *=* *13.07 DF = 26.60	*p* <0.0001
γ3	Control 0 dpe; Meng-Po OE 0 dpe	Not normal	Mann–Whitney test	14; 16	0.0; 99.3	*U *=* *14	*p* <0.0001
γ3	Control 7 dpe; Meng-Po OE 7 dpe	Not normal	Mann–Whitney test	12; 16	0.4; 291.8	*U *=* *0	*p* <0.0001
α’1	Control 0 dpe; *meng-po* RNAi 0 dpe	Not normal	Kruskal–Wallis test	10; 13	1.0; 0.5	Mean rank diff = 2.212	*p *>* *0.9999
α’1	Control 0 dpe; control 7 dpe	Not normal	Kruskal–Wallis test	10; 17	1.0; 31.7	Mean rank diff = −24.75	*p = *0.0004
α’1	Control 0 dpe; *meng-po* RNAi 7 dpe	Not normal	Kruskal–Wallis test	10 ; 16	1.0; 0.3	Mean rank diff = 6.125	*p *>* *0.9999
α’1	*meng-po* RNAi 0 dpe; control 7 dpe	Not normal	Kruskal–Wallis test	13; 17	0.5; 31.7	Mean rank diff = −26.96	*p < *0.0001
α’1	*meng-po* RNAi 0 dpe; *meng-po* RNAi 7 dpe	Not normal	Kruskal–Wallis test	13; 16	0.5; 0.3	Mean rank diff = 3.913	*p *>* *0.9999
α’1	Control 7 dpe; *meng-po* RNAi 7 dpe	Not normal	Kruskal–Wallis test	17; 16	31.7; 0.3	Mean rank diff = 30.88	*p < *0.0001
β’1	Control 0 dpe; *meng-po* RNAi 0 dpe	Not normal	Kruskal–Wallis test	10; 13	0.8; 0.3	Mean rank diff = 7.138	*p *>* *0.9999
β’1	Control 0 dpe; control 7 dpe	Not normal	Kruskal–Wallis test	10; 17	0.8; 41.7	Mean rank diff = −23.90	*p = *0.0007
β’1	Control 0 dpe; *meng-po* RNAi 7 dpe	Not normal	Kruskal–Wallis test	10 ; 16	0.8; 0.6	Mean rank diff = 4.194	*p *>* *0.9999
β’1	*meng-po* RNAi 0 dpe; control 7 dpe	Not normal	Kruskal–Wallis test	13; 17	0.3; 41.7	Mean rank diff = −31.04	*p < *0.0001
β’1	*meng-po* RNAi 0 dpe; *meng-po* RNAi 7 dpe	Not normal	Kruskal–Wallis test	13; 16	0.3; 0.6	Mean rank diff = −2.945	*p *>* *0.9999
β’1	Control 7 dpe; *meng-po* RNAi 7 dpe	Not normal	Kruskal–Wallis test	17; 16	41.7; 0.6	Mean rank diff = 28.09	*p < *0.0001
α’1	*shibire^ts^* 7 dpe 20°C; 25°C	Normal	Brown–Forsythe, Welch ANOVA test	15; 14	7.5; 25.5	*t *=* *3.74 DF = 16.00	*p *=* *0.0053
α’1	*shibire^ts^* 7 dpe 20°C; 33°C	Normal	Brown–Forsythe, Welch ANOVA test	15; 17	7.5; 62.2	*t *=* *11.90 D*F* =20.01	*p *<* *0.0001
α’1	*shibire^ts^* 7 dpe 25°C; 33°C	Normal	Brown–Forsythe, Welch ANOVA test	14; 17	25.5; 62.2	*t *=* *5.844 DF = 28.31	*p *<* *0.0001
β’1	*shibire^ts^* 7 dpe 20°C; 25°C	Not normal	Kruskal–Wallis test	16; 14	18.8; 25.6	Mean rank diff = −3.808	*p *>* *0.9999
β’1	*shibire^ts^* 7 dpe 20°C; 33°C	Not normal	Kruskal–Wallis test	16; 17	18.8; 66.9	Mean rank diff = −24.08	*p *<* *0.0001
β’1	*shibire^ts^* 7 dpe 25°C; 33°C	Not normal	Kruskal–Wallis test	14; 17	25.6; 66.9	Mean rank diff = −20.27	*p = *0.0001
α2	*shibire^ts^* 7 dpe 20°C; 25°C	Normal	Brown–Forsythe, Welch ANOVA test	16; 13	11.1; 53.8	*t *=* *9.33 DF = 16.03	*p* <0.0001
α2	*shibire^ts^* 7 dpe 20°C; 33°C	Normal	Brown–Forsythe, Welch ANOVA test	16; 17	11.1; 9.3	*t *=* *0.67 DF = 30.76	*p = *0.8747
α2	*shibire^ts^* 7 dpe 25°C; 33°C	Normal	Brown–Forsythe, Welch ANOVA test	13; 17	53.8; 9.3	*t *=* *9.53 DF = 17.13	*p *<* *0.0001
γ1	*shibire^ts^* 7 dpe 20°C; 25°C	Not normal	Kruskal–Wallis test	16; 14	1.9; 0.9	Mean rank diff = 4.353	*p *>* *0.9999
γ1	*shibire^ts^* 7 dpe 20°C; 33°C	Not normal	Kruskal–Wallis test	16; 17	1.9; 101.8	Mean rank diff = −21.47	*p *<* *0.0001
γ1	*shibire^ts^* 7 dpe 25°C; 33°C	Not normal	Kruskal–Wallis test	14; 17	0.9; 101.8	Mean rank diff = −25.82	*p *<* *0.0001
α2	*shibire^ts^* 7 dpe 20°C; 7 dpe 20°C, 3 h 33°C	Not normal	Mann–Whitney test	30; 26	3.6; 26.6	*U *=* *82.50	*p* <0.0001
α’1	UAS-TNT 0 dpe; UAS-TNT 7 dpe	Not normal	Mann–Whitney test	32; 57	1.2; 72.4	*U *=* *0	*p* <0.0001
β’1	UAS-TNT 0 dpe; UAS-TNT 7 dpe	Not normal	Mann–Whitney test	32; 57	0.5; 75.2	*U *=* *3.50	*p *<* *0.0001
γ1	UAS-TNT 0 dpe; UAS-TNT 7 dpe	Not normal	Mann–Whitney test	31; 57	0.6; 18.5	*U *=* *165.50	*p *<* *0.0001
α'3	UAS-TNT 0 dpe; UAS-TNT 7 dpe	Not normal	Mann–Whitney test	29; 54	0.3; 30.1	*U *=* *154	*p *<* *0.0001
α3	UAS-TNT 0 dpe; UAS-TNT 7 dpe	Not normal	Mann–Whitney test	20; 49	0.2; 66.8	*U *=* *0	*p *<* *0.0001
β’2m	UAS-TNT 0 dpe; UAS-TNT 7 dpe	Not normal	Mann–Whitney test	31; 57	0.6; 44.1	*U *=* *0	*p *<* *0.0001
β2	UAS-TNT 0 dpe; UAS-TNT 7 dpe	Not normal	Mann–Whitney test	32; 58	0.2; 85.1	*U *=* *0	*p *<* *0.0001
α’1	Control; UAS-TNT 7 dpe	Not normal	Mann–Whitney test	68; 57	35.9; 72.4	*U *=* *451.50	*p *<* *0.0001
β’1	Control; UAS-TNT 7 dpe	Normal	Unpaired two-tailed *t* test	72; 57	70.4; 75.2	*t *=* *0.83 df = 75.77	*p = *0.41
α’1 and β’1	α’1 UAS-TNT 7 dpe; β’1 UAS-TNT 7 dpe	Normal	Unpaired two-tailed *t* test	57; 57	72.4; 75.2	*t* =0.43 df = 101.59	*p *=* *0.67

## Results

### Age-dependent and sex-dependent PKA activity in MB lobe subfields

The MB consists of two bilateral KC clusters that project into five distinct axonal lobes; α/β, α’/β’, and γ (Fig. 1*A*). These lobes are further subdivided into discrete output neuron fields (Aso et al., 2014b). KCs can innervate multiple contiguous fields, which can have opposing behavioral outputs, raising the question of how multineuron interactions are regulated. PKA signaling required for learning and memory occurs at differential levels across the MB lobes (Skoulakis et al., 1993; Crittenden et al., 1998), suggesting local PKA activity regulation could drive developmental and circuit-level differences. To test PKA activity in defined MBON fields (Fig. 1*A*), we employed the Gal4/UAS system to drive expression of the UAS-PKA*-*separation of phases-based activity reporter of kinase (UAS-PKA-SPARK; Zhang et al., 2018) using pan-KC driver OK107-Gal4 (Connolly et al., 1996; Duffy, 2002; Aso et al., 2009). This reporter is phosphorylated specifically by PKA (Zhang et al., 2018), and PKA-SPARK generates reversible GFP puncta that can be imaged using confocal microscopy. PKA-SPARK::GFP puncta can be fixed and immunolabeled for co-labeling in MB studies (Sears and Broadie, 2020). Brains were dissected from staged male and female animals at timed dpe at 25°C, and immediately fixed for PKA-SPARK::GFP imaging. Representative MB lobe images and quantified results are shown in Figure 1.

PKA-SPARK signaling was compared at two time points: immediately after eclosion (0 dpe; Fig. 1*B*) and at maturity (7 dpe; Fig. 1*C*) following the early-use critical period, a developmental window when sensory input experience remodels MB circuitry (Dombrovski and Condron, 2021). In the absence of signaling, diffuse GFP labels the MB lobes, allowing mapping of the MBON fields (Fig. 1*A*,*B*). At 0 dpe, there are rarely detectable PKA-SPARK::GFP puncta in the MB lobes (Fig. 1*B*). In contrast, mature 7 dpe animals display strong PKA activity, with many puncta localized to distinct MBON fields (Fig. 1*C*). The α/β lobes have inconsistent, broadly distributed puncta. Prominent signaling is tightly restricted to α’1 (left arrow) and β’1 (right arrow) fields, with lower levels in the β’2ap field (Fig. 1*C*). Quantification in the α’1 field by counting PKA-SPARK::GFP puncta shows essentially no signaling at 0 dpe (0.1 ± 0.04, *n* = 38) compared with high levels of signaling at 7 dpe (32.5 ± 2.4, *n* = 34), a very significant increase over the short, one-week critical period (*p *<* *0.0001; Fig. 1*D*). Likewise, in the β’1 field, there is essentially no detectable PKA activity at the immature 0 dpe time point (0.1 ± 0.05, *n* = 37) compared with strong signaling at 7 dpe maturity (66.8 ± 3.3, *n* = 38), again a highly significant elevation (*p *<* *0.0001; Fig. 1*E*). β’1 displays ∼2-fold more PKA activity compared with α’1. These data show a dramatic increase in PKA signaling activity between 0 and 7 dpe, tightly restricted to a few MBON fields.

At 7 dpe, the number of PKA-SPARK::GFP puncta in the MB lobes is quite variable (Fig. 1*D*,*E*). We hypothesized that this variability could be attributable in part to sex-specific differences, with sexually dimorphic levels of PKA signaling in the MB α’/β’ lobes. Comparing males and females, there is no detectable change in immature stages (0 dpe), with both sexes showing little or no PKA activity. In contrast, there is a clear sex-dependent difference in PKA-SPARK::GFP puncta number at maturity (7 dpe) with females showing higher activity (Fig. 1*F*) compared with males (Fig. 1*G*). In both sexes, prominent PKA signaling is restricted to the α’1 and β’1 fields (arrows). In the α’1 field, females display nearly twice as many active puncta (50.1 ± 3.1, *n* = 18) compared with the males (26.9 ± 1.8, *n* = 16), a highly significant sex difference (*p *<* *0.0001; Fig. 1*H*). Similarly in the β’1 region, females have more PKA-SPARK::GFP puncta (83.2 ± 3.0, *n* = 18) compared with males (64.5 ± 3.6, *n* = 16), again a very significant sex-dependent difference *(p *<* *0.0001; Fig. 1*I*). Importantly, while weak PKA signaling can be detected in additional MB lobe regions in both females and males, there are no other consistent sex-specific differences outside of the striking PKA-SPARK::GFP puncta differences present in α’1 and β’1, with lower levels in the β’2ap MBON field. These data show adult-onset PKA activity in select MB lobe connectivity regions in both sexes, with females displaying comparatively more PKA signaling.

**Figure 2.**
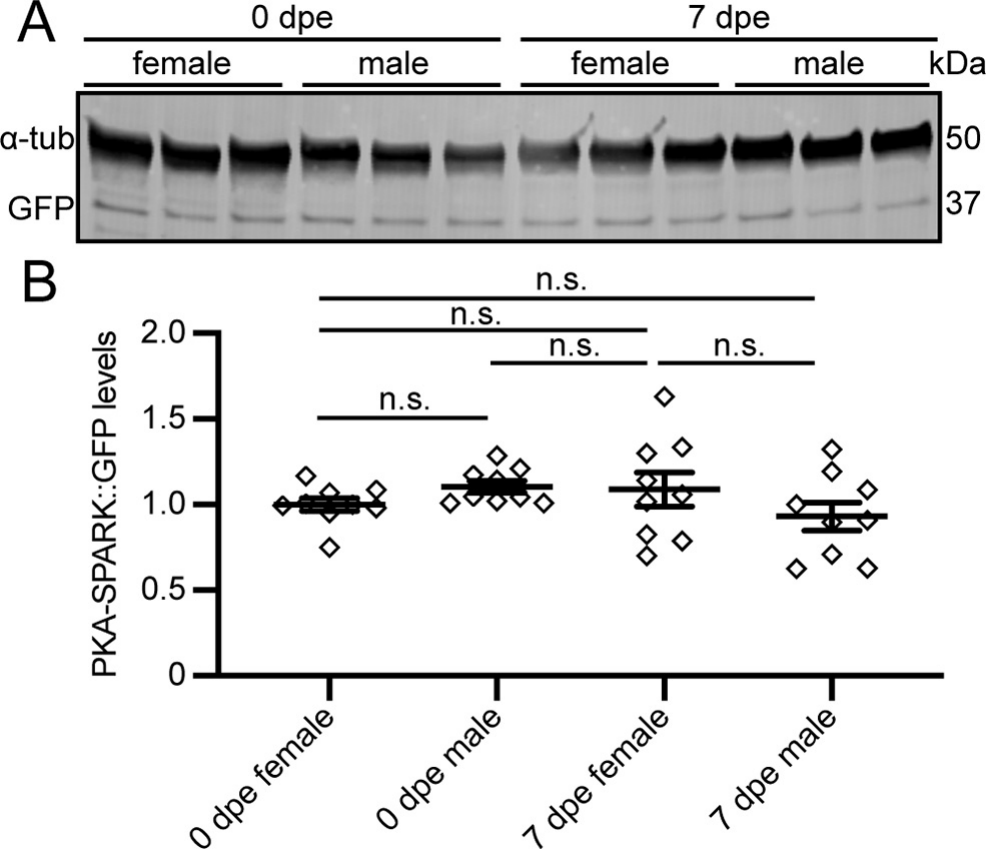
PKA-SPARK::GFP reporter levels constant across sex and age groups. ***A***, Representative Western blotting comparing PKA-SPARK::GFP protein levels (anti-GFP) from 0 and 7 dpe time points, in both females and males with OK107-Gal4 driving UAS-PKA-SPARK::GFP. The protein loading control is α-Tubulin (α-tub). Probed proteins are indicated on the left and molecular weights on the right. ***B***, PKA-SPARK::GFP protein levels normalized to the α-tub loading control. Scatter plots show all data points and mean ± SEM. Statistics show Brown–Forsythe and Welch ANOVA tests. Sample size: 9, all conditions. Significance: not significant (n.s.; *p *>* *0.05).

To determine whether the striking age-dependent and sex-dependent differences may be because of expression level changes of the PKA-SPARK biosensor, we performed Western blot analysis of adult head lysates (Fig. 2). As above, the MB-specific OK107-Gal4 driver was used to express the PKA-SPARK::GFP reporter. Developmentally staged heads (0 and 7 dpe) were collected for both sexes. For each of the four groups (either female or male, and either 0 or 7 dpe), two heads per sample were pooled, processed and assayed in parallel for GFP levels relative to the α-tubulin loading control (Fig. 2). A single GFP-specific band was detected at ∼37 kDa (Fig. 2*A*), corresponding to the expected size of the GFP-containing LRRATLVD-EGFP-HOTag3 reporter construct (Zhang et al., 2018). In all trials, the reporter levels were indistinguishable between both ages (0 and 7 dpe) and sexes (female and male; Fig. 2*A*). In the quantified comparisons, PKA-SPARK::GFP levels were highly comparable between all groups, including both ages and sexes (normalized to 0 dpe female 1.0 ± 0.04; 0 dpe male 1.10 ± 0.03; 7 dpe female 1.09 ± 0.10; 7 dpe male 0.93 ± 0.08; *n* = 9 for each group; *p *>* *0.05 for all comparisons; Fig. 2*B*). Taken together, these data show that the changes in PKA signaling are not because of reporter expression differences, but represent striking age-specific and sex-specific PKA activity levels within the MB lobes.

### Localized MB lobe PKA activity driven by FMRP and Rugose

**Figure 3.**
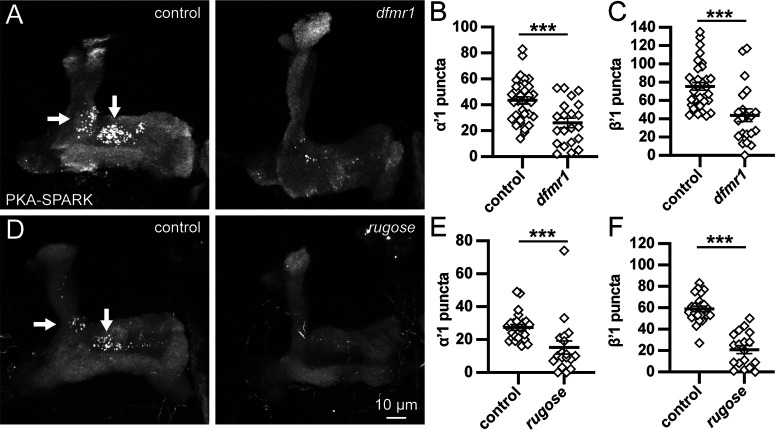
Localized MB lobe PKA activity signaling enabled by FMRP and Rugose. ***A***, Representative images of MB lobes with OK107-Gal4 driving UAS-PKA-SPARK at 7 dpe in control (left) and *dfmr1^50M^* null (right). Arrows point to α’1 and β’1 (Fig. 1*A*). PKA-SPARK::GFP puncta number in α’1 (***B***) and β’1 (***C***) regions. ***D***, Similar confocal imaging comparison at 7 dpe in control (left) and *rg^FDD^* null (right). ***E***, ***F***, Quantification of PKA-SPARK::GFP puncta number in α’1 (***E***) and β’1 (***F***) regions. Note that this comparison is done in males only owing to the X chromosome location of the *rugose* gene. Scatter plots show all data points and mean ± SEM. Sample size >12 fields in all conditions. Statistics show two-tailed *t* tests with Welch’s correction (***B***, ***C***, ***F***) or Mann–Whitney tests (***E***). Significance: ***p <* *0.001.

We next sought to determine the molecular mechanisms for the spatiotemporal restriction of PKA activity signaling in the MB lobes. We first tested disease models causally linked to PKA misregulation and impaired MB-dependent learning/memory. FMRP is involved in cAMP induction and PKA activation (Berry-Kravis et al., 1995; Sears and Broadie, 2020). In the *Drosophila* FXS disease model of intellectual disability, *dfmr1* null mutants exhibit severely defective PKA-dependent learning/memory based on MB signaling dysfunction (Bolduc et al., 2008). The RNA-binding translational regulator FMRP binds directly to Rugose/NBEA mRNA to drive expression of this PKA anchor within KCs (Sears et al., 2019). In a second *Drosophila* disease model of intellectual disability, *rugose* (*rg*) null mutants also display impaired MB-dependent learning and memory (Wang et al., 2000; Volders et al., 2012). Based on these studies, we hypothesized the FMRP-Rg pathway regulates the spatiotemporal restriction of PKA activity signaling in the MB lobes (α’1 and β’1; Fig. 1). To test this idea, we compared PKA-SPARK analyses in the genetic background control (*w^1118^*) to both the *dfmr1^50M^* (Zhang et al., 2001) and *rg^FDD^* (Volders et al., 2012) null alleles. Assays of staged animals tested differences within the defined MBON fields. Representative images and quantified data are shown in Figure 3.

At 7 dpe, *w^1118^* controls show striking active PKA-SPARK::GFP puncta formation primarily restricted to the MB lobe α’1 and β’1 fields (Fig. 3*A*, arrows). In stark contrast, *dfmr1^50M^* nulls display a severe reduction in PKA signaling activity in both of these regions (Fig. 3*A*, right). Quantification of the α’1 region reveals far more puncta in the controls (43.5 ± 2.8, *n* = 34) compared with *dfmr1^50M^* mutants (26.1 ± 3.7, *n* = 21), a highly significant loss of signaling (*p *=* *0.0005; Fig. 3*B*). Likewise in the β’1 region, there are many more PKA-SPARK::GFP puncta in controls (75.5 ± 4.2, *n* = 34) compared with *dfmr1^50M^* nulls (43.9 ± 7.0, *n* = 21), showing a significant reduction in the localized PKA activity (*p *=* *0.0004; Fig. 3*C*). Similarly, in *rg^FDD^* null mutants, there is an obvious loss of signaling in both α’1 and β’1 MBON fields (Fig. 3*D*). Quantification of the α’1 field shows many more puncta in the controls (27.4 ± 1.8, *n* = 24 males only) compared with *rg^FDD^* mutants (15.2 ± 4.0, *n* = 18), a very significant reduction (*p *=* *0.0001; Fig. 3*E*). Moreover, in the β’1 field, there is nearly 3-fold more signaling in the controls (58.9 ± 2.6, *n* = 24) compared with *rg^FDD^* nulls (20.8 ± 3.7, *n* = 18), a highly significant loss of signaling (*p *<* *0.0001; Fig. 3*F*). As in controls, few PKA-SPARK::GFP puncta appear outside of these regions. These data indicate that both FMRP and Rugose are similarly required to enable PKA activity in the MB lobes, but do not alter the spatiotemporal restriction of the PKA signaling domains.

### Spatiotemporal PKA activity bidirectionally regulated by Meng-Po kinase

**Figure 4.**
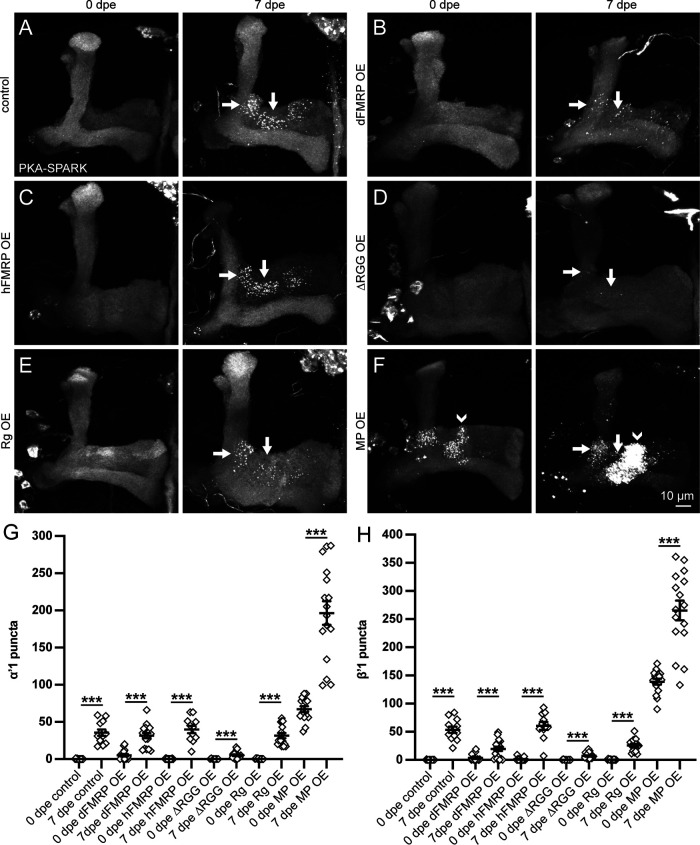
Localized circuit PKA activity increased with Meng-Po OE. ***A–F***, Representative images of MB lobes with OK107-Gal4 driving UAS*-*PKA-SPARK at 0 and 7 dpe in control (***A***) and with targeted OE of UAS-dFMRP (***B***), UAS-hFMRP (***C***), UAS-ΔRGG-hFMRP (***D***), UAS-Rugose (Rg, ***E***), and UAS-Meng-Po (MP, ***F***). Arrows indicate the α’1 and β’1 regions (Fig. 1*A*) of normally heightened PKA activity. Arrowhead (***F***) points to the expanded PKA activity within the γ3 region. ***G***, ***H***, Quantification of PKA-SPARK::GFP puncta in α’1 (***G***) and β’1 (***H***) regions. Scatter plots show all data points and mean ± SEM. Sample size: >7 fields in every genotype and at every time point. Statistics show two-tailed *t* tests with Welch’s correction and Mann–Whitney tests (see statistical table; Table 1). Significance: ***p <* *0.001.

Given the tightly localized PKA activity, we next tested OE effects on spatiotemporal misregulation. Driving both *Drosophila* FMRP (dFMRP) and human FMRP (hFMRP) elevates PKA activity in KC somata (Sears and Broadie, 2020). We hypothesized expanded or enhanced PKA activity in the MB lobes by OE of the FMRP-Rg regulatory pathway. Using OK107-Gal4 to drive UAS-PKA-SPARK, we tested UAS-dFMRP (Zhang et al., 2001), UAS-hFMRP (Sears and Broadie, 2020), UAS-ΔRGG-hFMRP (Coffee, 2011), and UAS-Rg (Volders et al., 2012). ΔRGG-hFMRP OE localizes PKA activity to phase-separated domains in KC somata and proximal process, but the impact on lobe signaling has not previously been tested (Sears and Broadie, 2020). We also assayed OE of Meng-Po (MP) kinase, which synergizes with PKA activity in the MB (Lee et al., 2018). Meng-Po localizes to the MB lobes and UAS-MP OE dramatically strengthens MB-dependent learning and memory (Lee et al., 2018). We hypothesized enhanced spatiotemporal PKA activity in the MB lobes through increased Meng-Po function. For all of these OE comparisons, we visualized PKA-SPARK::GFP puncta throughout the MB lobes at both immature 0 dpe and mature 7 dpe time points. The MB driver OK107-Gal4 with UAS-PKA-SPARK alone was the reference control. Representative images at both time points and the quantified data for controls and all five OE conditions are shown in Figure 4.

FMRP-Rg pathway OE did little to promote PKA activity at 0 dpe, with low signaling in all conditions (Fig. 4*A–E*). Quantification of PKA-SPARK::GFP puncta in the α’1 field shows controls (0.1 ± 0.1, *n* = 14) is similar to both hFMRP OE (0.2 ± 0.1, *n* = 18, *p *>* *0.05) and ΔRGG-hFMRP OE (0.1 ± 0.1, *n* = 8, *p *>* *0.05), albeit with a slight increase following dFMRP OE (5.2 ± 0.6, *n* = 16, *p *=* *0.03; Fig. 4*G*). Elevated Rugose did not increase PKA activity (0.3 ± 0.1, *n* = 14, *p *>* *0.05). In β’1, there is also little early signaling increase at 0 dpe (Fig. 4*A–E*). Quantification shows similarly low puncta in controls (0.1 ± 0.1, *n* = 14), hFMRP OE (1.0 ± 0.6, *n* = 18, *p *>* *0.05), ΔRGG-hFMRP OE (0.1 ± 0.1, *n* = 8, *p *>* *0.05) and Rg OE (0.4 ± 0.2, *n* = 14, *p *>* *0.05), with slight elevation after dFMRP OE (3.4 ± 1.5, *n* = 16, *p *=* *0.12; Fig. 4*H*). Like controls, there is PKA activity upregulation at 7 dpe in all OE conditions, but no extra increase and no expansion outside α’1, β’1, and β’2ap regions (Fig. 4*A–E*). In α’1, PKA-SPARK::GFP puncta are similar in controls (35.7 ± 4.3, *n* = 12) and dFMRP OE (31.6 ± 3.5, *n* = 18, *p *>* *0.05), hFMRP OE (39.8 ± 5.1, *n* = 11, *p *>* *0.05) and Rg OE (31.6 ± 3.5, *n* = 16, *p *>* *0.05), albeit lower in ΔRGG-hFMRP OE (5.3 ± 1.1, *n* = 18, *p *< 0.0001; Fig. 4*G*). In β’1, puncta are similar in controls (53.6 ± 5.5, *n* = 12) and hFMRP OE (60.5 ± 7.1, *n* = 11), but reduced with dFMRP OE (19.6 ± 3.7, *n* = 18, *p *=* *0.0002), Rg OE (25.7 ± 2.8, *n* = 16, *p *=* *0.0014) and especially ΔRGG-hFMRP OE (7.1 ± 1.2, *n* = 18, *p < *0.0001; Fig. 4*H*).

Meng-Po (MP) OE causes a profound induction of PKA signaling, strongly subverting normal temporal restrictions (compare Fig. 4*A* and *F*). At immature 0 dpe, MP induces striking PKA activity in the normal α’1 and β’1 regions, which is not yet present in controls, and expands high signaling to the γ3 field (Fig. 4*F*, arrowhead). In α’1, the nearly undetectable PKA-SPARK::GFP puncta in controls (0.1 ± 0.1, *n* = 14) are highly elevated by Meng-Po OE (67.1 ± 3.9, *n* = 16), a very significant increase (*p *<* *0.0001; Fig. 4*G*, right). Likewise, in β’1, the absence of puncta in controls (0.1 ± 0.1, *n* = 14) is even more highly increased by Meng-Po OE (138.9 ± 5.2, *n* = 16, *p *<* *0.0001; Fig. 4*H*). In the newly-recruited γ3 field, there are also a large number of active puncta (control 0.0 ± 0.0, *n* = 14; Meng-Po OE 99.3 ± 18.7, *n* = 16, *p < *0.0001). This elevated and expanded PKA signaling is more pronounced at maturity (7 dpe), with higher activity in α’1, β’1, and γ3 MBON fields (Fig. 4*F*, right). Quantification in the α’1 field shows PKA-SPARK::GFP puncta increased >2-fold by Meng-Po OE (196.4 ± 15.9, *n* = 16), a very significant increase (*p *<* *0.0001; Fig. 4*G*, right). Likewise, in β’1, puncta are similarly elevated with Meng-Po OE (251.9 ± 14.2, *n* = 22, *p *<* *0.0001; Fig. 4*H*). In the new γ3 activity field, there is dramatic PKA signaling (291.8 ± 17.7, *n* = 16) that is absent in controls (0.4 ± 0.2, *n* = 12, *p *< 0.0001). These data show that gain of Meng-Po function strongly expands spatiotemporal PKA activity.

**Figure 5.**
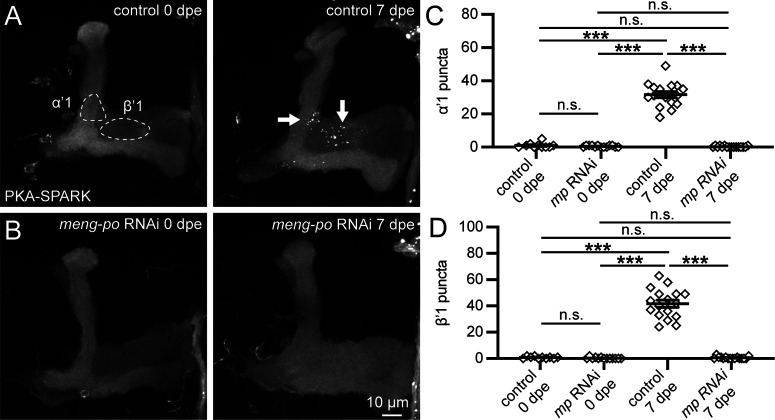
Localized MB lobe PKA activity signaling requires the Meng-Po kinase. ***A***, Representative MB lobe images with OK107-Gal4 driving UAS-PKA-SPARK at both 0 dpe (left) and 7 dpe (right) in the genetic control background. Quantified MBON fields (dashed circles, left) and arrows indicate α’1 and β’1 regions. ***B***, Representative MB lobes images with *meng-po* RNAi under the same conditions as in ***A***. Scale bar: 10 μm. ***C***, ***D***, Quantification of PKA-SPARK::GFP puncta in α’1 (***C***) and β’1 (***D***) MBON fields. Scatter plots show all data points and mean ± SEM. Statistics show Kruskal–Wallis tests. Sample size: >9, all conditions. Significance: ***p <* *0.001, not significant (n.s.; *p *>* *0.05).

The strong effect of Meng-Po OE suggests a requirement promoting PKA activity, consistent with its role as a PKA-synergist within the MB (Lee et al., 2018). To test this hypothesis, we next assayed MP LOF. In our hands, *meng-po* mutants are lethal before adult eclosion, so MB-specific *meng-po* RNAi was tested for changes in MB lobe PKA activity (Fig. 5). To test for spatiotemporal changes in PKA signaling, the RNAi knock-down was compared with the driver alone transgenic control at both 0 and 7 dpe. Similar to the controls at 0 dpe, there are very few or no detectable PKA-SPARK puncta observed in the *meng-po* RNAi animals (Fig. 5*A*,*B*, left panels). In contrast, mature 7 dpe controls exhibit strong PKA activity, again mostly localized to the α’1 and β’1 regions (Fig. 5*A*, right), whereas the *meng-po* RNAi animals exhibit no detectable PKA-SPARK puncta (Fig. 5*B*, right). Quantification of the α’1 field shows high puncta numbers only in the controls at 7 dpe (control 0 dpe 1.0 ± 0.5, *n* = 10; *meng-po* RNAi 0 dpe 0.5 ± 0.1, *n* = 13; control 7 dpe 31.7 ± 1.8, *n* = 17; *meng-po* RNAi 7 dpe 0.3 ± 0.1, *n* = 16; *p *<* *0.001 between control 7 dpe and all others groups, *p *>* *0.05 for all other comparisons; Fig. 5*C*). Similarly, β’1 PKA-SPARK puncta are only elevated in controls at 7 dpe (control 0 dpe 0.8 ± 0.2, *n* = 10; *meng-po* RNAi 0 dpe 0.3 ± 0.2, *n* = 13; control 7 dpe 41.7 ± 2.7, *n* = 17; *meng-po* RNAi 7 dpe 0.6 ± 0.2, *n* = 16; *p *<* *0.001 between control 7 dpe and other groups, *p *>* *0.05 for all other comparisons; Fig. 5*D*). Taken together, these data show Meng-Po promotes localized PKA activity signaling in the MB lobes.

### Conditional *shibire^ts^* neurotransmission block increases PKA activity

**Figure 6.**
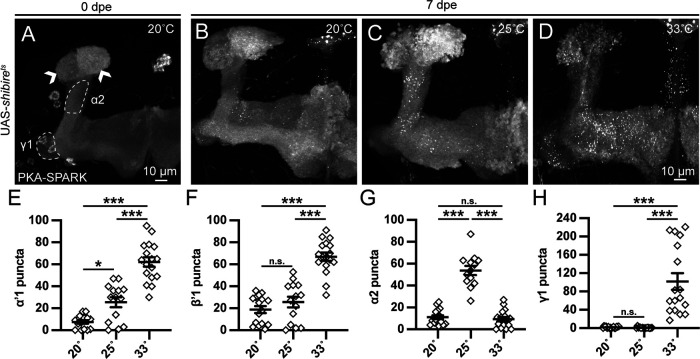
Conditional *shibire^ts^* neurotransmission block increases PKA activity. ***A–D***, Representative images of MB lobes with OK107-Gal4 driving UAS*-shibire^ts^* at the 0 and 7 dpe time points at the indicated adult rearing temperatures. Dotted outlines define additional MBON regions of elevated PKA activity detected with the PKA-SPARK reporter, and arrowheads indicate expanded α/α’ neuropils. ***E–H***, Quantified number of PKA-SPARK:GFP puncta in defined MB lobe regions. Scatter plots show all data points and the mean ± SEM. Sample size >12 fields in every temperature condition. Statistics show Brown–Forsythe and Welch ANOVA tests (***E***, ***G***) and Kruskal–Wallis tests (***F***, ***H***). Significance: *p <* *0.05, ***p <* *0.001 and not significant (n.s.; *p *>* *0.05).

The spatiotemporally localized PKA activity within a few MBON fields suggests local network regulation. Multiple circuit feedback interactions are proposed to provide modularity to the MB circuit, both within and between signaling compartments (Liu and Davis, 2009; Inada et al., 2017; Modi et al., 2020). We hypothesized blocking KC synaptic output could alter PKA activity signaling measured by PKA-SPARK::GFP puncta localization. To test effects of output connectivity on PKA activity, we used the temperature-sensitive *shibire^ts^* allele (Koenig et al., 1983; Kitamoto, 2001), which is frequently employed to block neurotransmission (Jacob et al., 2021; Muria et al., 2021). At restrictive temperatures (>29°C), *shibire^ts^* is defective in SV recycling, and semi-dominant over wild type (Koenig et al., 1983; Kitamoto, 2001). At reported permissive temperature (25°C) some phenotypes manifest, so we also added a 20°C condition. Using the MB-targeted OK107-Gal4 driver to express PKA-SPARK, *shibire^ts^* animals were reared at 20°C until 0 dpe, then aged for 7 d at 20°C, 25°C or 33°C. Restrictive temperature *shibire^ts^* animals at 0 dpe display little to no PKA-SPARK::GFP puncta throughout the MB lobes (Fig. 6*A*), but have enlarged α/α’ ends (arrowheads). The *shibire^ts^* animals reared to 7 dpe display variable levels of PKA activity with altered MB lobe spatial patterns depending on temperature (20–33°C). Representative images at all temperatures and quantified data are shown in Figure 6.

Mutants reared at low temperature to 0 dpe (20°C), already show some altered MB morphology, including enlarged α/α’ lobe ends (Fig. 6*A*, arrows). With rearing at 20°C to 7dpe, there are fewer PKA-SPARK::GFP puncta in α’1 and β’1 regions, with a low density also in β’2ap (Fig. 6*B*). At 25°C, there is higher PKA signaling in different MB lobe regions (Fig. 6*C*). At restrictive 33°C, *shibire^ts^* display greatly elevated PKA activity in both α’1 and β’1, as well as β’2ap (Fig. 6*D*). Quantification in α’1 shows the fewest PKA-SPARK::GFP puncta at 20°C (7.5 ± 1.6, *n* = 15), more at 25°C (25.5 ± 4.6, *n* = 14), and the most at 33°C [62.2 ± 4.3, *n* = 17 (Fig. 6*E*), 20° vs 25° *p *=* *0.0053, 20° vs 33° *p *<* *0.0001, 25° vs 33°, *p *<* *0.0001]. Similarly, in β’1, there are increasing puncta from 20°C (18.8 ± 3.2, *n* = 16) to 25°C (25.6 ± 4.9, *n* = 14) to 33°C [66.9 ± 3.7, *n* = 17 (Fig. 6*F*), 20° vs 25° *p *>* *0.05, 20° vs 33° *p *< 0.0001, 25° vs 33° *p *=* *0.0001]. At 25°C, there is also more α2 PKA activity, while at 33°C signaling is expanded across the β’/γ lobes (Fig. 6*D*). To quantify this expanded signaling, α2 and γ1 regions were assayed. In α2, few puncta at 20°C (11.1 ± 1.7, *n* = 16) are increased at 25°C (53.8 ± 4.2, *n* = 13), but not at 33°C [9.3 ± 2.0, *n* = 17 (Fig. 6*G*), 20° vs 25° *p *<* *0.0001, 20° vs 33° *p *>* *0.05, 25° vs 33°, *p *<* *0.0001]. In γ1, there is no difference between 20°C (1.9 ± 0.4, *n* = 16) and 25°C (0.9 ± 0.4, *n* = 14, *p *>* *0.05), but a very significant increase at restrictive 33°C (101.8 ± 18.3, *n* = 17, *p *<* *0.0001; Fig. 6*H*). More acute *shibire^ts^* blockade (3 h at 33°C) also increases PKA signaling. The largest elevation in PKA-SPARK puncta number is in α2; a ∼7-fold increase compared with the 20°C control (20°C 3.6 ± 0.7, *n* = 30; 33°C for 3 h 26.6 ± 4.6, *n* = 26; *p *<* *0.0001). These findings show that *shibire^ts^* impairment of KC neurotransmission greatly broadens PKA activity in the MB lobes.

### PKA signaling activity dramatically expanded by synaptic output block

**Figure 7.**
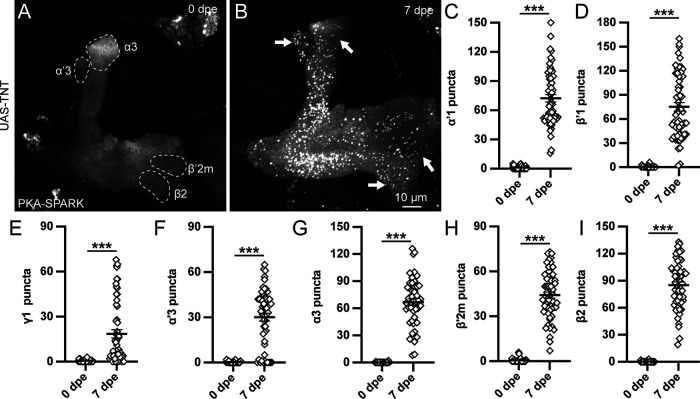
MB circuit PKA activity dramatically expanded by synaptic output block. ***A***, ***B***, MB lobes with OK107-Gal4 driving UAS*-tetanus toxin light chain* (TNT) at both 0 dpe (***A***) and 7 dpe (***B***). Arrows indicate expanded PKA activity regions detected with the PKA-SPARK reporter and dotted outlines indicate newly-recruited MBON regions. ***C–I***, Quantification of PKA-SPARK::GFP puncta number in each defined MBON region, including α’1 (***C***), β’1 (***D***), γ1 (***E***), α’3 (***F***), α3 (***G***), β’2m (***H***), and β2 (***I***). Scatter plots show all data points and the mean ± SEM. Sample size >19 fields in every paired comparison. Statistics show Mann–Whitney tests. Significance: ***p <* *0.001.

The above *shibire^ts^* studies suggest that MB synaptic output suppresses PKA activity across MBON fields, but interpretation is complicated by temperature and pleiotropic Dynamin functions. We therefore turned to a stronger, synapse-selective neurotransmission block via targeted transgenic tetanus toxin light chain (UAS-TNT; Sweeney et al., 1995) to silence synapses (Chow et al., 2011; Haag et al., 2016; Fuenzalida-Uribe and Campusano, 2018). The TNT protease cleaves the v-SNARE Synaptobrevin to block SV exocytosis and thus presynaptic function (Link et al., 1992; Schiavo et al., 1992). Using MB-targeted OK107-Gal4 to drive UAS-TNT, PKA-SPARK animals were reared at 25°C until 0 and 7 dpe. Synaptically-silenced MBs display normal morphology (Fig. 7*A*,*B*), confirming that KC neurotransmission can be blocked without affecting MB lobe architecture. Within the defined MBON fields, PKA activity was tested by assaying PKA-SPARK::GFP puncta. TNT-blocked animals at 0 dpe continue to display very few PKA-SPARK::GFP puncta throughout the MB lobes (Fig. 7*A*), demonstrating that blocked KC synaptic output does not alleviate the temporal restriction on PKA signaling. In contrast, however, TNT-blocked animals at 7 dpe display greatly expanded PKA activity, with PKA-SPARK::GFP puncta appearing widely throughout the MB lobes (Fig. 7*B*). Representative images and quantified data from multiple MBON fields are shown in Figure 7.

In newly-eclosed animals (0 dpe), the TNT blockade of KC synaptic transmission has no effect within any MBON field (Fig. 7*C–I*), with very few or no active PKA-SPARK::GFP puncta detectable in quantification trials. In sharp contrast, mature TNT-blocked animals (7 dpe) exhibit a profound increase in PKA activity signaling throughout MB lobe regions, with especially prominent puncta numbers present in both α/β and α’/β’ lobes (Fig. 7*B*). Interestingly, while widespread ectopic γ lobe PKA activity is also frequently observed, it is not universally present in TNT-blocked animals. The α’1 and β’1 regions display numerous activated PKA-SPARK::GFP puncta, with a greater relative elevation in the α’1 region compared with the β’1 region (Fig. 7*B*). With the quantification of PKA-SPARK::GFP puncta in TNT-blocked animals, α’1 activity is >2-fold increased (72.4 ± 3.8, *n* = 57; Fig. 7*C*) relative to controls (35.9 ± 1.8, *n* = 68, *p *<* *0.0001), but there is little change in the β’1 MBON field (control: 70.4 ± 2.2, *n* = 72; UAS*-TNT* 75.2 ± 5.3, *n* = 57; Fig. 7*D*). This striking shift shows a balance change in activity-dependent PKA signaling. Under control conditions, there is always consistently higher numbers of PKA-SPARK::GFP puncta in β’1 compared with α’1 (Fig. 1*D*,*E*). However, in TNT-blocked animals these regions are no longer statistically different (*p *>* *0.05; Fig. 7*C*,*D*). These data suggest that blocking KC neurotransmission disrupts the network signaling balance between MBON fields.

TNT synaptic-silencing of KC output generates strongly elevated PKA signaling in widespread MBON fields that normally lack any detectable PKA activity (Fig. 7*B*). In TNT-blocked animals, PKA-SPARK::GFP puncta are now prominently distributed across the MB lobes (arrows). To quantify this elevated PKA signaling, puncta counts were performed in the γ1, α’3, α3, β’2m, and β2 regions (Fig. 7*E–I*). None of these MBON fields normally show detectable PKA activity (Fig. 1*C*). In TNT-blocked animals, the lack of PKA-SPARK::GFP puncta in the γ1 region at 0 dpe (0.6 ± 0.14, *n* = 31) is replaced with significantly elevated numbers by 7 dpe (18.5 ± 2.8, *n* = 57, *p *<* *0.0001; Fig. 7*E*). Likewise, in α’3, TNT blockade does not increase PKA activity at 0 dpe (0.3 ± 0.1, *n* = 29), but induces many puncta by 7 dpe (30.1 ± 2.6, *n* = 54, *p *<* *0.0001; Fig. 7*F*). The adjacent α3 is also similarly inactive at 0 dpe (0.2 ± 0.1, *n* = 20), and highly active at 7 dpe (66.8 ± 3.9, *n* = 49, *p *<* *0.0001; Fig. 7*G*). The smaller β’2m region displays similar striking induction (0 dpe: 0.6 ± 0.6, *n* = 31; 7 dpe: 44.1 ± 2.1, *n* = 57, *p *<* *0.0001), as does the adjacent β2 region (0 dpe: 0.2 ± 0.1, *n* = 32; 7 dpe: 85.1 ± 3.4, *n* = 58, *p *<* *0.0001; Fig. 7*H*,*I*). Taken together, these data suggest that MB lobe regional field-selective PKA activity levels are regulated by experience-driven mechanisms in the young adult, and that widespread PKA activity in the MB lobes is suppressed by neurotransmission-dependent network feedback.

## Discussion

In this study, we explore the spatiotemporal regulation of PKA activity within the MB lobes. PKA signaling initiates in early adulthood, with heightened activity in just 3/16 MB lobe output neuron fields (α’1, β’1, and β’2ap; Aso et al., 2014b; Modi et al., 2020). In addition to age-dependence, this regional PKA signaling displays sex-dependence, with elevation in females over males (Fig. 1). These findings were made possible with the PKA-SPARK biosensor; this fluorescent reporter uses motifs found in earlier PKA sensors, with pharmacological and genetic approaches promoting and preventing PKA activity verifying this new tool in cell culture and *in vivo* (Zhang et al., 2018; Sears et al., 2019; Tyurin-Kuzmin et al., 2020). While it is possible that the PKA-SPARK reporter is revealing only the strongest PKA activity (Zhang et al., 2018), at the least α’1, β’1, and β’2ap connectivity regions have much higher PKA activity levels compared with the rest of the MB neuropil. In two disease models of intellectual disability and ASDs, from loss of either FMRP or Rugose/NBEA (Zhang et al., 2001; Volders et al., 2012), PKA activity remains spatiotemporally restricted, but is dramatically reduced (Fig. 3). There is surprisingly little change from overactivation of the FMRP→Rg control pathway (Sears et al., 2019), but PKA activity is profoundly altered by the PKA pathway Meng-Po kinase (Lee et al., 2018), with OE enhancing spatiotemporal PKA signaling (Fig. 4) and loss suppressing PKA activity (Fig. 5). Network feedback downstream of KC neurotransmission strongly suppresses PKA activity, since blocking KC synaptic output with conditional *shibire^ts^* (Koenig et al., 1983; Kitamoto, 2001) or transgenic TNT (Sweeney et al., 1995; Haag et al., 2016) induces widespread PKA activity signaling (Figs. 6, 7). Thus, localized PKA activity is highly regulated at the circuit level.

At a macro level, the α’1 and β’1 regions with the highest localized PKA activity levels (Fig. 1) have been linked to valence (Aso et al., 2014a), i.e., whether local activation causes animals to approach or avoid a stimulus. The α’1 and β’2 fields exhibit opposing output valence (positive and negative, respectively), while the β’1 role appears less clear (Aso et al., 2014a; Modi et al., 2020). However, higher PKA signaling can be activated in the γ3 region (via Meng-Po; Fig. 4), which in combination with the β’1 region drives positive valence (Aso et al., 2014a). Moreover, dopaminergic and serotonergic biosensor signals function through the β’ region from a variety of external sense stimuli (Sun et al., 2018; Wan et al., 2021), to which the α’1/β’1 fields display high sensitivity (Inada et al., 2017), suggesting broad responsiveness. Very recent work shows high spontaneous activity in α’/β’ restricted to young animals (Leinwand and Scott, 2021), suggesting α’/β’ PKA signaling may be controlled by selective developmental activity. Moreover, inhibiting *miR-92a* in α/β and γ, but not α’/β’, was shown to enhance memory (Guven-Ozkan et al., 2020), indicating another layer of lobe-selective circuit regulation. Note that the output neurons from these MB lobe regions are different (α’1 cholinergic, β’1 GABAergic, and β’2 glutamatergic; Aso et al., 2014b), suggesting more complex integrative circuit functions. PKA signaling activation after the early-use critical period (Fig. 1) is consistent with sensory experience-dependent regulation (Dombrovski and Condron, 2021). Importantly, MB sensory integration functions differ markedly between females and males (Park et al., 2018; Shin et al., 2020), correlating with the report here of sex-dependent PKA signaling differences in females compared with males (Fig. 1).

Two learning and memory proteins, FMRP and Rugose, are needed for full PKA activity in the α’1 and β’1 MB lobe output neuron fields (Fig. 3). RNA-binding FMRP is a translational regulator (Darnell et al., 2011; Greenblatt and Spradling, 2018) known to facilitate PKA signaling (Berry-Kravis et al., 1995; Kelley et al., 2007), which is lost in the FXS, the commonest heritable cause of intellectual disability and ASD (Harris et al., 2008). Rugose/NBEA is a PKA-anchor that facilitates learning and memory, and is also associated with ASDs (Wang et al., 2000; Castermans et al., 2003, 2010; Volders et al., 2012). Previous work has shown FMRP binds to *rugose* mRNA to drive KC expression (Sears et al., 2019; Buddika et al., 2021). As predicted, disruption of this FMRP→Rg regulative pathway strongly impairs PKA activity in the MB lobes (Fig. 3). In contrast, localized PKA signaling is dramatically strengthened by MB OE of the Meng-Po kinase (Lee et al., 2018), which induces early-onset PKA activity before adult sensory experience, spatially expands high PKA activity to the γ3 MBON field, and profoundly elevates PKA activity within all the normal MB lobe regions of heightened PKA signaling (Fig. 4). Consistently, Meng-Po kinase OE also greatly improves learning and memory, via PKA phosphorylation, but additionally via signaling feedback synergy (Lee et al., 2018). Moreover, *meng-po* RNAi causes the opposite result of reducing localized PKA-SPARK puncta (Fig. 5). Based on both loss and gain of function, we suggest Meng-Po enhances localized PKA activity, reflecting circuit level kinase regulation. Determining how Meng-Po-regulated PKA activity determines circuit excitability and regional balance will be a major subject of future research.

Two different KC synaptic output blocking methods dramatically expand PKA activity signaling in the MB lobes (Figs. 6, 7). Both conditional *shibire^ts^* and transgenic tetanus toxin tools block KC neurotransmission, but through quite different mechanisms (Kitamoto, 2001; Haag et al., 2016). At 33°C, KC-targeted *shibire^ts^* drives PKA activity expansion in the MB γ lobe (Fig. 6). This change could indicate cross-compartment network interactions between the γ lobe and other MB regions. Increasing γ1 PKA activity is especially interesting, as γ1 toggles inhibition of other MB regions (Perisse et al., 2016). The tetanus toxin protease blocks neurotransmission through eliminating SV exocytosis (Sweeney et al., 1995; Haag et al., 2016), and therefore provides a stronger and more selective means to silence KC synaptic output. Consistently, TNT animals show a more profound expansion of PKA activity throughout the MB lobes, albeit again affecting only spatial and not temporal patterning (Fig. 7). Neither *shibire^ts^* nor TNT blockade alters early PKA activity (Figs. 6, 7), suggesting induction of PKA signaling is determined primarily by later experience-driven activity. Localized PKA activity changes with KC output block implies active circuit balance; for example, weighing aversive versus attractive responses to sensory stimuli (Modi et al., 2020). The widespread PKA activity upregulation with KC output block leads us to hypothesize that enhancing KC neuron activity should result in elevated PKA signaling.

At the MB circuit level, multiple candidate synaptic pathways need to be explored for roles in local PKA activity regulation in different MB lobe output neuron fields. GABAergic inputs to dopaminergic neurons are one likely candidate, since GABA treatment has been shown to correct *dfmr1* mutant circuit defects exacerbated by glutamate exposure (Chang et al., 2008; Yamagata et al., 2021). Moreover, GABAergic anterior paired lateral (APL) neurons broadly control MB activity through feedback to the KCs, and are most strongly activated by the α’/β’ lobes (Liu and Davis, 2009; Lin et al., 2014; Inada et al., 2017; Modi et al., 2020). Recent work shows that treatment with a dopamine transport inhibitor also ameliorates *rg* mutant social interaction and memory deficits (Pham et al., 2021). Another candidate is the Amnesiac neuropeptide from the serotonergic dorsal paired medial neurons, required for their normal development in the broad innervation of the MB lobes (Yu et al., 2005; Lee et al., 2011; Turrel et al., 2018). In the context of our studies, *de facto* depression may feedback onto KCs to promote PKA activity signaling. The impact of upstream input onto the MB lobes is an important consideration, including how this circuitry combines with spontaneous MB activity and internal lobe circuitry to determine PKA signaling. Future research directions should attempt to dissect how these different layers of neuromodulation control localized PKA activity signaling within the MB lobe circuit, and between females and males, by manipulating input-specific neuronal activity in targeted transgenic studies.

In conclusion, we report here that PKA activity signaling in the *Drosophila* brain MB learning and memory center is highly induced during early experiential adulthood, with selective upregulation in the α’1, β’1, and β’2ap MB lobe output neuron regions. Age-specific and sex-specific PKA signaling controlled within KCs and downstream of KC output shows that spatiotemporally restricted MB lobe PKA activity is regulated through a combination of both intracellular control and intercellular network-level mechanisms. Importantly, PKA signaling can be precociously promoted and spatial expanded though the activity of the PKA-synergist Meng-Po kinase. Moreover, KC neurotransmission inhibits localized PKA signaling within the MB circuit. Our future studies will be aimed toward generating new genetic responder tools to test KC signaling with both neurotransmission output blockade and activity promotion of upstream and downstream MB circuit components, simultaneously and independently of KCs. We also plan to test PKA activity signaling with the manipulation of specific KC partners, by altering neurotransmission signaling in combination with postsynaptic neurotransmitter receptor mutants to determine network communication cues. Taken together with the current work, these ongoing studies will continue to expand our understanding of circuit-level PKA signaling regulation in normal function and in neurologic disease model contexts.

## Synthesis

Reviewing Editor: Michael Michaelides, NIDA-NIH Decisions are customarily a result of the Reviewing Editor and the peer reviewers coming together and discussing their recommendations until a consensus is reached. When revisions are invited, a fact-based synthesis statement explaining their decision and outlining what is needed to prepare a revision will be listed below. The following reviewer(s) agreed to reveal their identity: NONE.

Reviewer 1

Studies in Drosophila contributed to the idea that PKA is very important in consolidating learning and memory by finding that learning and memory mutants in genes such as dunce encoded components of the cAMP / PKA pathway. High-level expression of several of these genes in the Drosophila mushroom bodies (MBs) identified MBs as the likely site of learning and memory, and more recent advances in understanding the connectivity of MBs have identified the relevant output areas of MBs for memory-driven behavior. In short, studying the Drosophila MBs is an excellent opportunity to study learning-driven synaptic plasticity. However, PKA activity has been difficult to visualize until fairly recently when the PKA-SPARK reporter was generated. This adds to the growing toolkit of sensors to monitor neuronal activity, Ca2+ levels etc.

In this manuscript, the authors take a first step in this direction by using the PKA-SPARK reporter at different ages of flies and in different mutant backgrounds. Overall, the data are well-presented and I only have a few specific comments described below. In addition, the data are very clear that PKA-SPARK puncta are increased between day 0 and day 7, are decreased in Fmrp and Neurobeachin (rugose) mutants, and are increased by Meng Po overexpression. It is also very clear that TNT expression in MBs in broadly increases PKA-SPARK puncta numbers across the MBs, indicating that MB-MB signaling - either directly or indirectly via feedback from another neuronal type - normally keeps PKA activity low in various MB regions.

Major comments:

1. The data showing more PKA-SPARK puncta in old vs young MBs and in female vs male flies is very clear. Can the authors exclude the more trivial possibility that there is higher expression of PKA-SPARK at these fly ages or in females vs males?

2. For non-MB experts, how did the authors determine which region of the MB shows PKA-SPARK puncta? Is this purely anatomical or was there a co-stain for MB regions?

3. Why did the authors not complement their gain-of-function Meng Po data with loss-of-function data? Did Meng Po mutants not show a phenotype, or are the mutants not viable and therefore the experiment is not possible to perform? It would be interesting to know.

4. The authors kept the shibire flies at 33 for 7 days. Why did they inactivate MB signaling for so long? It would have been much more interesting to see if there is a short-term effect of MB signaling on PKA activity. Given the TNT data, I am not sure how useful this experiment is and perhaps it needs a better rationale.

5. The discussion is quite repetitive of the data and is much longer than is necessary for a clear paper like this.

Minor comments:

1. Please explain “early-use critical period” for a more general audience.

2. I would prefer that the numerical data (e.g. 53.58{plus minus}5.47, n=12) be moved to the figure legend because it makes it harder to read the text. This is even worse in the description of Figure 4 where the paragraph seems to have more numbers than words. It also seems too precise to present the numbers of puncta to 2 decimal places when puncta are presumably counted as a whole number.

Very minor comments:

1. Add some white space between graphs D and E, and H and I in Figure 1. Alternatively, move the y-axis label closer to the y-axis numbers in E and I to make it easier to see which graph is which.

Reviewer 2

The manuscript entitled “Temporally and Spatially Localized PKA Activity within Learning and Memory Circuitry Regulated by Network Feedback” shows that PKA activity in a learning and memory circuit is both age-dependent and sex-dependent. Overall, I found the findings interesting and thought the experiments were well-conducted. Below are a few things that I think could strengthen the manuscript.

1. Is PKA activity reduced in Meng Po Kinase loss-of-function mutants? Given that this experiment was done with FMRP and Rugose mutants, I think it should also be done for Meng Po Kinase mutants.

2. Does PKA activity change in KCs if synaptic activity is increased or decreased in neurons that innervate KCs?

3. Can the authors provide more information on why PKA-SPARK is a reliable indicator of PKA activity in the introduction or discussion. All of the findings in the manuscript are with this biosensor, so I think more information should be provided.

## Author Response

Dear Editor,

We resubmit here our invited eN-NWR-0450-21 revision for your February 20 deadline. Below, are point-by-point responses to the referee comments. This revision includes two new figures, as well as additional experiments and text changes recommended by the referees. We followed all the journal guidelines for publication, but please let us know if anything further is needed.

Reviewer 1:

The Reviewer writes “the data are well-presented and I only have a few specific comments” and “the data are very clear”. We address the numbered comments below:

Major comments:

1. The data showing more PKA-SPARK puncta in old vs young MBs and in female vs male flies is very clear. Can the authors exclude the more trivial possibility that there is higher expression of PKA-SPARK at these ages, or in females vs. males?

To address these questions, we did PKA-SPARK Western blot analyses comparing both ages and sexes. We found no differences between any of these groups, indicating that the reported age- and sex-specific changes are not due to altered reporter expression. These new findings are included in a new figure (new Figure 2) and new Results paragraph (page 11, paragraph 2).

2. For non-MB experts, how did the authors determine which region of the MB shows PKA-SPARK puncta? Is this purely anatomical or was there a co-stain for MB regions?

The PKA-SPARK reporter expression provides the signal to determine mushroom body regions.

3. Why did the authors not complement their gain-of-function Meng Po data with loss-of-function data? Did Meng Po mutants not show a phenotype, or are the mutants not viable and therefore the experiment is not possible to perform? It would be interesting to know.

The Reviewer is correct; we were unable to collect Meng Po loss-of-function mutants and therefore could not perform this experiment. However, we have now performed experiments using Meng Po RNAi to show a loss of PKA activity signaling. These new findings are included in a new figure (new Figure 5) and new Results paragraphs (page 16, paragraph 2).

4. The authors kept the shibire flies at 33 for 7 days. Why did they inactivate MB signaling for so long? It would have been much more interesting to see if there is a short-term effect of MB signaling on PKA activity. Given the TNT data, I am not sure how useful this experiment is and perhaps it needs a better rationale.

To address short-term effects, we tested shibire animals at 33˚C for 3 hours. Consistent with the longer temperature shift, we found increased PKA-SPARK puncta. The new findings are shown in the Results (page 18, paragraph 1). As for the rationale, while the TNT data are compelling, the shibire data are consistent and provide an independent means of confirming the results.

5. The discussion is quite repetitive of the data and is much longer than is necessary for a clear paper like this.

We have streamlined the Discussion by removing repetitive sentences and speculation.

Minor comments:

1. Please explain “early-use critical period” for a more general audience.

We have added a sentence to the second paragraph of the first Results section (page 9).

2. I would prefer that the numerical data be moved to the figure legend because it makes it harder to read the text. This is even worse in the description of Figure 4 where the paragraph seems to have more numbers than words. It also seems too precise to present the numbers of puncta to 2 decimal places when puncta are presumably counted as a whole number.

As suggested, we have reduced numbers to one decimal place, which streamlines the text. We have kept numerical data in the text, following the eNeuro guidelines. We also prefer this format.

Very minor comments:

1. Add some white space between graphs D and E, and H and I in Figure 1. Alternatively, move y-axis labels closer to y-axis numbers in E and I to make it easier to see which graph is which.

As suggested, we have followed the first suggestion by adding more white space in Figure 1.

Reviewer 2:

The Reviewer writes “I found the findings interesting and thought the experiments were well-conducted. Below are a few things that I think could strengthen the manuscript.”

1. Is PKA activity reduced in Meng Po Kinase loss-of-function mutants? Given that this experiment was done with FMRP and Rugose mutants, I think it should also be done for Meng Po Kinase mutants.

As discussed above, we were unable to collect viable adult Meng Po loss-of-function mutants. Therefore, we used Meng Po RNAi to show loss of PKA signaling. These new findings are included in a new figure (new Figure 5) and Results paragraphs (page 16, paragraph 2)).

2. Does PKA activity change in KCs if synaptic activity is increased or decreased in neurons that innervate KCs?

As suggested, we are planning to pursue this question in future studies. However, this work is far beyond the scope of a revision. The complexity of using a dual-expression system, coupled with contributions from multiple inputs (e.g. GABA, serotonin, dopamine), makes for a long-term study. We have added more on this point to the Discussion section (page 27, paragraph 1).

3. Can the authors provide more information on why PKA-SPARK is a reliable indicator of PKA activity in the introduction or discussion. All of the findings in the manuscript are with this biosensor, so I think more information should be provided.

As suggested, we have added additional information and reference citations to the Discussion (page 23, paragraph 1).
